# Unveiling the Antioxidant Role of Hemp Oils in Cancer Prevention and Treatment

**DOI:** 10.3390/cancers17132128

**Published:** 2025-06-25

**Authors:** Marios C. Christodoulou, Panagiotis Rodosthenous, Christiana M. Neophytou

**Affiliations:** 1 Laboratory of Chemical Engineering and Engineering Sustainability, Faculty of Pure and Applied Sciences, Open University of Cyprus, 2231 Nicosia, Cyprus; 2 Department of Chemistry, Faculty of Pure and Applied Sciences, University of Cyprus, 2109 Nicosia, Cyprus; prodos02@ucy.ac.cy; 3 Department of Physics, Faculty of Pure and Applied Sciences, University of Cyprus, 2109 Nicosia, Cyprus; 4 Department of Life Sciences, European University Cyprus, 2404 Nicosia, Cyprus

**Keywords:** cancer, full-spectrum hemp oil, broad spectrum hemp oil, CBD oil

## Abstract

The global incidence of cancer continues to rise at an alarming rate, with annual cases projected to increase by 47% from 19.3 million in 2020 to 28.4 million by 2025. *Cannabis sativa* L. was among the earliest plants investigated for potential anticancer therapies, due to its more than 100 bioactive constituents that confer notable antioxidant properties. Hemp-derived extracts, particularly those rich in cannabidiol (CBD), exhibit notable synergistic biological effects, including the inhibition of cancer cell proliferation, angiogenesis, and metastasis, alongside the promotion of apoptosis. These pharmacological attributes suggest that hemp oils may serve as promising alternatives or adjuncts to conventional chemotherapy, offering potential therapeutic benefits with a reduced risk of severe adverse effects. This review discusses the current literature on hemp oils, with emphasis on their roles in cancer prevention, therapeutic efficacy, and potential toxicity in humans. Furthermore, it explores the various extraction methods employed in hemp oil production and examines their chemical compositions, offering a comprehensive understanding of the principal antioxidant constituents responsible for their bioactivity to the readers.

## Simple Summary

Cancer is strongly associated with oxidative stress induced by free radicals, which damage cellular components, leading to genetic mutations, the disruption of normal cellular functions, and the promotion of carcinogenesis. Hemp oils, which are rich in natural antioxidants such as cannabinoids, flavonoids, and terpenes, have been proposed as potential mediators to lessen oxidative stress and inhibit cancer progression by neutralizing free radicals and modulating biological pathways involved in cancer development. This review presents a comprehensive analysis of the antioxidant and anticancer properties of hemp oils, with a particular focus on their potential role in cancer prevention and treatment in humans. It also addresses extraction techniques, chemical composition, therapeutic applications, and the potential toxicological risks associated with their use.

## 1. Introduction

Cancer comprises a group of diseases characterized by dysregulated cell proliferation and the distribution of abnormal cells throughout the body. It can arise in nearly any organ or tissue within the body when cells begin to grow uncontrollably, forming tumors or metastasizing through the bloodstream and lymphatic system to other regions [1]. This pathological process is frequently triggered by genetic mutations and environmental factors, which lead to the formation of free radicals [2].

While at low concentrations free radicals contribute crucially to cellular signaling and defense mechanisms of our bodies, elevated levels may induce oxidative stress, which is deeply implicated in carcinogenesis, mutation, and cellular transformation [3,4]. In addition to this, it has been proven that certain types of cancer are more closely associated with oxidative stress and free radical damage than others [3,5,6]. Specifically, lung cancer is strongly linked to oxidative stress due to smoking and air pollution, both of which generate high levels of reactive oxygen species (ROS) and reactive nitrogen species (RNS) that damage DNA and promote carcinogenesis [7,8,9]. Examples include the formation of superoxide (O_2_^−^·), hydrogen peroxide (H_2_O_2_), hydroxyl (OH·), and nitric oxide (NO·) free radicals [4]. Similarly, ROS and RNS produced by UV radiation can cause tandem mutations in the p53 gene, resulting in different forms of skin cancer [10,11]. Colon cancer is also associated with chronic inflammation and oxidative stress within the gastrointestinal tract, where inflammatory conditions create a microenvironment rich in ROS, fostering DNA damage and cancer cell proliferation [8,12]. Furthermore, prostate and bladder cancers also show significant correlations with oxidative stress, evidenced by elevated biomarkers of oxidative damage and altered antioxidant enzyme levels in patients [13,14].

The precise origins of free radicals remain incompletely understood, though ongoing research continues to elucidate their sources and mechanisms. As far as we know, free radicals can be formed in two ways: exogenously or endogenously (with the help of enzymes or through non-enzymatic reactions). Exogenous sources include prolonged exposure to ultraviolet (UV) radiation from sunlight, X-rays, industrial and agricultural chemicals, and heavy metals, as well as diets high in processed foods, sugars, and unhealthy fats [3,4]. Additionally, psychological and physical stress, chronic infections, excessive physical exertion, alcohol consumption, active and passive smoking, and certain drug treatments can further induce free radical formation within the human body [8,11]. On the other hand, endogenous enzymatic reactions, which serve as sources of ROS, include those involved in the respiratory chain, phagocytosis, prostaglandin synthesis, Nicotinamide Adenine Dinucleotide Phosphate (NADPH) oxidases, and the cytochrome P-450 system, while non-enzymatic endogenous sources are primarily linked to mitochondrial, peroxisomal, xanthine oxidoreductase, and arachidonate pathway processes [5,11].

Additionally, the autoxidation of polyunsaturated fatty acids in humans leads to the formation of peroxyl radicals (ROO·), as lipid hydroperoxides (LOOH) can decompose in the presence of transition metals such as iron (Fe^2+^), resulting in the formation of alkoxyl radicals (RO·) and hydroxyl radicals (OH·) [4,15]. Likewise, RNS are formed through the activity of nitric oxide synthase enzymes, which convert L-arginine into nitric oxide (NO·) and L-citrulline. Once NO· is produced, it can react with O_2_^−^· to form ONOO^−^, a highly reactive molecule. Peroxynitrite can further decompose into other reactive species, such as OH· and nitrogen dioxide (NO_2_·) [8].

Human bodies naturally generate endogenous antioxidant enzymes, such as superoxide dismutase (SOD), catalase (CAT), and glutathione peroxidase (GPx), to defend against free radical formation. In addition, radical-scavenging antioxidants, such as vitamin C and E, can neutralize radicals to prevent the initiation and propagation of oxidation chains, serving as a secondary defense mechanism. This process may also involve the termination of a chain reaction through the interaction of two radicals [6]. However, these defense mechanisms are often insufficient in preventing extensive cellular damage. Therefore, the imbalance between free radicals and antioxidants leads to oxidative stress [6,16].

The human diet plays a critical role in maintaining the body’s antioxidant defense system by providing essential minerals that act as cofactors for the synthesis and function of key antioxidant enzymes. Furthermore, it enhances overall antioxidant capacity through the intake of various bioactive compounds, including vitamins, phenols, flavonoids, phenolic acids, and carotenoids [6]. These natural antioxidants are commonly found in a variety of plant-based sources, including roots, fruits, vegetables, nuts, seeds, herbs, oils, and spices [17,18,19]. Among these, hemp-derived oils, such as full-spectrum or broad spectrum hemp oils, hemp seed oil, hemp essential oil, and cannabidiol (CBD) oil, have emerged as some of the most sought-after products worldwide, owing to their potential therapeutic properties, including antioxidant, anticancer, and anti-inflammatory effects [20,21]. These benefits are primarily attributed to their rich composition of bioactive compounds, including cannabinoids, terpenes, flavonoids, and other phytonutrients derived from the *Cannabis Sativa* L. plant.

In particular, extracts rich in CBD, the primary non-intoxicating compound of the *Cannabis* plant, have been extensively investigated for their therapeutic potential in the treatment of various conditions, including epilepsy [22], sleep and neurodegenerative disorders [23], and management of pain [24,25]. Moreover, preclinical research suggests that CBD may address many of the pathways involved in the pathogenesis of cancers [26]. The potential benefits of CBD are further augmented by its co-occurrence with other bioactive constituents naturally present in *Cannabis Sativa* extracts, including Δ^9^-tetrahydrocannabinol (Δ^9^-THC), the plant’s main psychoactive cannabinoid compound. Additional synergy is provided by terpenes, flavonoids, and various other phytonutrients, which are believed to interact through the “entourage effect”, enhancing the overall pharmacological activity [27]. However, the uncontrolled daily consumption of Cannabis-infused edibles with high concentrations of CBD is not without risk [28]. According to the U.S. Food and Drug Administration (FDA), while CBD shows therapeutic promise, data on its long-term safety remains limited. The agency cautions that such products may pose significant health risks. Documented adverse effects include hepatotoxicity, potential interactions with concurrently administered medications, gastrointestinal issues such as diarrhea and appetite fluctuations, and mood changes including increased irritability [29].

Building upon this foundation, the present review aims to synthesize and critically evaluate the current body of scientific literature concerning the therapeutic potential and safety profile of hemp oils that are commercially available, with particular emphasis on its application in cancer prevention and treatment in humans. For this purpose, data were retrieved from three major academic databases, Scopus, PubMed, and Google Scholar, encompassing publications from the year 2000 through May 2025. In addition, it provides a detailed examination of the extraction methodologies utilized for various *Cannabis* oil types and explores their antioxidant and anticancer mechanisms. Crucially, the analysis also identifies several research gaps, including a lack of robust clinical validation, and inadequate standardization of hemp oil formulations, all of which warrant further investigation. To the best of our knowledge, no comprehensive review article to date has been published that concurrently addresses all of the aforementioned aspects.

## 2. Carcinogenesis

The process of carcinogenesis starts through a series of genetic and epigenetic alterations, resulting in uncontrolled cell proliferation. The process is typically divided into three stages. The first stage, initiation, involves genetic mutations or DNA alterations caused by factors such as chemicals, viruses, or spontaneous errors in DNA replication [1]. Additionally, DNA damage can occur due to radiation. When radiation interacts with water molecules in cells, it leads to the production of hydroxyl radicals and other reactive species through a process called radiolysis. These hydroxyl radicals predominantly interact with DNA by adding to the double bonds of pyrimidine bases or by abstracting hydrogen from the sugar moieties [5]. Radiation can also directly hit the DNA molecules, causing breaks or alterations in the DNA structure. These mutations can accumulate and potentially lead to various biological effects, including carcinogenesis.

When cells incur DNA damage, the p53 gene, which is a crucial tumor suppressor, acts as a surveillance mechanism, slowing cell cycle progression and initiating apoptosis [9]. The activation of p53 is highly associated with increased production of ROS. In lung cancer, p53 is frequently mutated, impairing its ability to induce apoptosis. Mutant p53 often accumulates in the cytoplasm and functions as an oncogene. It is the most commonly altered gene in human lung cancer, with a mutation rate of 50–55%, particularly in squamous cell carcinoma and small-cell carcinoma of the lung [30]. These mutations are primarily G to T transversions caused by DNA adduct formation with carcinogens like polycyclic hydrocarbons, commonly found in smokers’ lungs [31].

Moreover, certain polymorphisms in genes related to oxidative stress play a crucial role in carcinogenesis by promoting mutations and tumor growth. The human genome contains approximately 10 million single nucleotide polymorphisms (SNPs). While many of these SNPs are silent, some in coding or regulatory regions result in phenotypic changes. SNPs in the cytochrome P450 (CYP) gene family, which includes phase I detoxifying enzymes and a phase II metabolic and antioxidant enzyme, are linked to the detoxification of environmental carcinogens and various human cancers [32]. In addition, SNPs in DNA repair genes, such as those involved in the repair of 8-hydroxy-2′-deoxyguanosine (8-OHdG) through base excision repair (BER) enzymes, as well as those involved in nucleotide excision repair (NER) pathways, can also contribute to carcinogenesis [10]. Likewise, epidemiological studies have identified SNPs in 8-oxo-guanine DNA glycosylase (OGG1) as being significantly associated with an increased risk of cancers such as lung, esophageal, prostate, and gastric cancers [33].

The second stage, promotion, is when the mutated cells are stimulated to divide and proliferate. This stimulation occurs under the influence of promoting agents like hormones, dietary factors, and chronic inflammation [1]. More specifically, hormones can act as growth signals, promoting the division of cells that have already acquired mutations, while components in the diet, like certain fats or lack of specific nutrients, can influence cell proliferation. Persistent inflammation can create an environment rich in growth factors and cytokines, which enhance cell division and survival, and facilitate processes such as angiogenesis, immune evasion, and metastasis [34].

In this stage, ROS and RNS contribute to carcinogenesis by modulating apoptotic pathways [35]. In particular, ROS induce intrinsic apoptosis by causing mitochondrial damage, which leads to the release of cytochrome C into the cytoplasm [36]. This process also results in the loss of the anti-apoptotic protein Bcl-2, degradation of mitochondrial mRNA, rRNA and DNA, and a reduction in mitochondrial genome transcription [9]. Additionally, ROS activate pro-apoptotic signaling molecules such as apoptosis signal-regulating kinase 1 (ASK1), c-Jun N-terminal kinase (JNK), and p38 [37]. It is also worth noting that highly invasive or metastatic cancer cells may require a certain level of oxidative stress to maintain a balance between proliferation and apoptosis. These cells generate large amounts of hydrogen peroxide that functions as a signaling molecule involved in cancer cell survival [10].

Similarly, RNS can have both pro-apoptotic and anti-apoptotic effects, depending on the cell type, cellular redox state, and nitric oxide radical concentration [38]. Although the precise apoptotic pathways and molecular targets of NO are not fully established, recent evidence suggests that NO’s pro-apoptotic activity involves the p38/MAPK pathway, mitochondrial death receptors, and GAPDH cell death mechanisms [39]. In addition, Activator Protein-1 (AP-1) and Nuclear Factor kappa-light-chain-enhancer of activated B-cells (NF-κB) are key transcription factors highly responsive to ROS and RNS. When exposed to oxidants and inflammatory cytokines such as TNF-α, both AP-1 and NF-κB are activated, leading to the modulation of gene expression [9]. AP-1, composed of proteins from the Fos, Jun, ATF, and MAF families, regulates genes involved in cell proliferation, differentiation, and apoptosis [40]. NF-κB, which is kept inactive in the cytoplasm by IκB proteins, translocates to the nucleus upon activation, influencing genes related to immune and inflammatory responses, as well as cell survival [41]. Dysregulation of AP-1 and NF-κB activation is implicated in chronic inflammatory conditions and cancer, as chronic activation promotes cell proliferation and survival while inhibiting apoptosis, thus facilitating tumorigenesis.

Lipid oxidation can also occur during the promotion stage. This process takes place in polyunsaturated fatty acids within cell membranes and progresses through a radical chain reaction. Initiated by hydroxyl radicals, lipid peroxidation commences with the abstraction of hydrogen atoms, resulting in the formation of lipid radicals, which subsequently convert into diene conjugates. When these lipid radicals interact with oxygen, they form peroxyl radicals. These highly reactive peroxyl radicals then attack other fatty acids, producing lipid hydroperoxides and new radicals, thereby perpetuating the lipid peroxidation process [5,15]. This chain reaction yields various biomarkers that can be tracked and monitored, including alkanes, malondialdehyde, isoprostanes, isofurans, or aldehydes [42].

Several proteins can also be oxidatively altered through three principal mechanisms: the oxidation of specific amino acids, peptide cleavage facilitated by free radicals, and the formation of protein cross-links due to interactions with lipid peroxidation products [5]. Different concentrations of ROS can trigger various signaling pathways. Low or transient ROS levels can stimulate proliferative signals, whereas high ROS levels typically inhibit cell proliferation by activating damage or cell death-related pathways. Moreover, research has shown that ROS can activate kinases and inactivate phosphatases, as well as activate protein serine/threonine kinases, small G proteins, and transcription factor signaling. These pathways are altered in different ways in cancer cells, leading to a loss of control over cell proliferation and differentiation [10]. In addition, amino acids such as methionine, cysteine, arginine, and histidine are particularly prone to oxidative damage. Such oxidative damage can impair the functional activities of enzymes, receptors, and membrane transport systems. Furthermore, the oxidative modification of proteins can result in changes to signal transduction pathways, enzyme activities, thermal stability, and increased susceptibility to proteolysis, all of which contribute to the aging process [5,16].

At the third stage of carcinogenesis, known as progression, proliferating cells accumulate additional genetic mutations, including mutations in oncogenes, tumor suppressor genes, and other regulatory genes, driving uncontrolled growth and division [1]. The genetic and epigenetic changes result in a more malignant phenotype, with increased cell proliferation, survival, resistance to cell death, and the ability to adapt to various environmental stresses [34]. Cancer cells gain the ability to invade surrounding tissues by producing enzymes that degrade the extracellular matrix (ECM) and base membrane, facilitating their movement into adjacent tissues. They develop mechanisms to evade the immune system, altering the expression of surface antigens, secreting immunosuppressive molecules, or recruiting regulatory immune cells to create an immunosuppressive microenvironment, helping them avoid detection and destruction by the body’s immune defenses [34,43].

**Figure 1:**
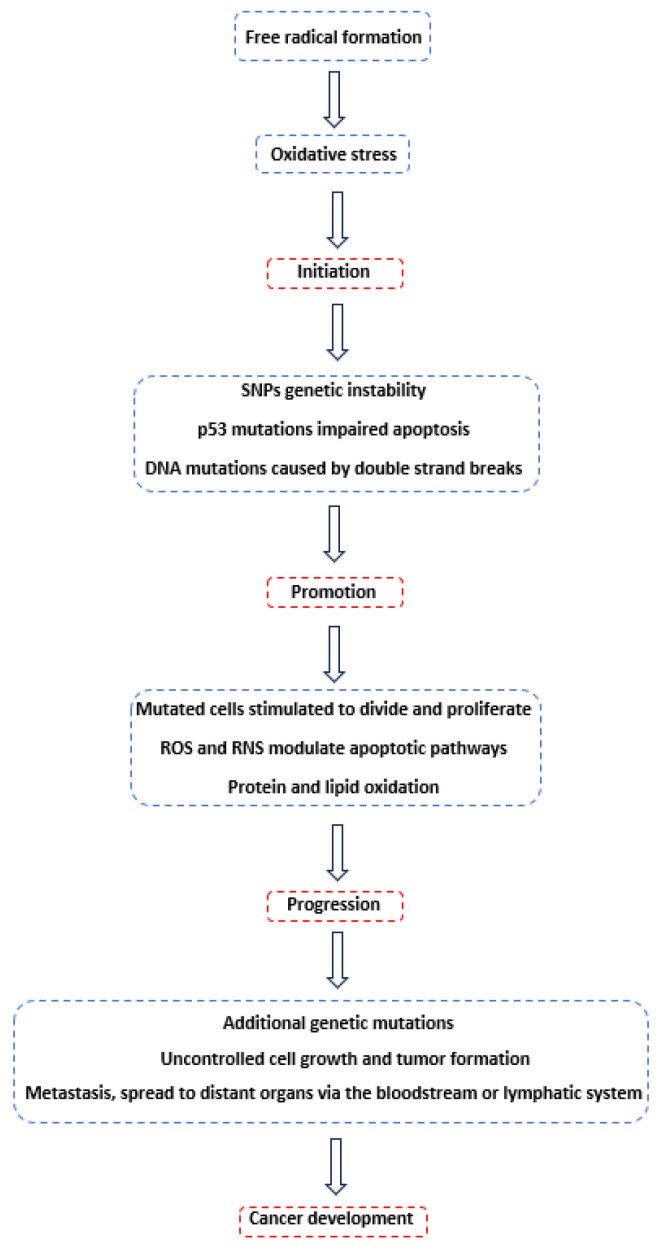
A flow chart summarizing the process of carcinogenesis, which comprises three primary stages: initiation, promotion, and progression. The process begins with free radical formation, advances through genetic mutations and the proliferation of mutated cells, and culminates in tumor formation and metastasis [1,5,9,32,34,44].

In the final stage of carcinogenesis, cancer cells acquire the ability to metastasize, enabling their spread to distant organs via the bloodstream or lymphatic system. The metastatic cascade involves several steps: cancer cells must undergo epithelial–mesenchymal transition, detach from the primary tumor, invade locally, intravasate into the circulatory and lymphatic systems, evade immune surveillance, extravasate at distant capillary beds, and invade and proliferate in distant organs, leading to the creation of secondary tumors. Several hypotheses have been proposed to elucidate the origins of cancer metastasis, including the accumulation of mutations in stem cells, macrophage facilitation processes, and a macrophage origin involving either transformation or fusion hybridization with neoplastic cells. However, the precise mechanisms remain incompletely understood [44]. An outline of the process of carcinogenesis is summarized in Figure 1.

## 3. Legislation for *Cannabis*-Based Edibles

*Cannabis* legislation is evolving globally, with many countries progressively broadening their regulatory frameworks to address not only the illicit and medicinal use of *Cannabis* but also its lawful incorporation into food products and cosmetic formulations [45]. Within this context, the European Commission has issued specific recommendations to its member states, urging the systematic monitoring of THC levels and other cannabinoids such as tetrahydrocannabinolic acid A (THCA-A), tetrahydrocannabinolic acid B (THCA-B), cannabinol (CBN), CBD, and tetrahydrocannabivarin (THCV) in *Cannabis*-infused consumables [46,47]. In addition to this, the Federal Institute for Risk Assessment (BfR) and the European Food Safety Authority (EFSA) have recommended an acute reference dose (ARfD) of 1 μg of Δ^9^-THC per kilogram of body weight per day to mitigate potential health risks associated with *Cannabis*-based products [47,48]. These regulatory limits apply to the combined content of Δ^9^-THC and its precursor THCA. Furthermore, in accordance with EU Recommendation 2016/2115, cannabinoid concentration in food products must be verified using analytical techniques based on mass spectrometry, thereby ensuring accuracy and compliance with safety standards [49]. At the same time, the Danish National Food Institute (DNFI) in 2018 established an ARfD of 0.4 μg/kg bw for children and adolescents. Moreover, DNFI stated that the provided values do not ensure sufficient protection of small children (<4 years old) and discouraged hemp-based food consumption in those ages [50].

Despite the latter regulations, the latest dietary Δ^9^-THC exposure assessment conducted by EFSA showed that ARfD of 1 μg/kg bw for total THC was exceeded at the upper bound (UB) for adult consumers in all food categories, whereas the largest exposure was characteristic for tea, beer, and beer-like beverages, as well as hemp seed oil [49]. This is due to the fact that many EU countries, including Cyprus, Belgium, Germany, Denmark, and Italy, have established their own stricter regulatory thresholds for THC in food products. As shown in Table 1, the maximum allowable limit for THC in *Cannabis*-based edibles varying significantly. The table is further expanded to include regulatory frameworks from the United States and Canada, Switzerland, New Zealand, and Australia although it is important to note that legislation may vary across individual states or provinces within these countries [28,46,47,51,52,53,54,55,56,57,58,59,60,61].

Notably, Belgium allows up to 10 mg/kg of THC in hemp seed oil, 5 mg/kg in hemp seeds and flour, 0.04 mg in soft drinks, and 0.2 mg in other food and beverage products [64]. Similarly, Germany has established a provisional daily intake limit for THC of 1–2 µg/kg bw. Based on this threshold, it recommends that consumers avoid beverages (both alcoholic and non-alcoholic) containing more than 5 µg/kg of THC, edible oils exceeding 5000 µg/kg, and other food products with THC levels surpassing 150 µg/kg [47,62]. In this context, since 2022, Cyprus has implemented a zero-tolerance policy for THC in all types of *Cannabis*-infused edible products, while the sale of CBD products requires a special license issued by the appropriate regulatory authorities [65].

Switzerland issued maximum levels for Δ^9^-THC of 20 mg/kg in hemp seed oil, 10 mg/kg for hemp seeds, 5 mg/kg for spirits (based on pure alcohol), 2 mg/kg for bakery and long-life bakery products and pasta, 1 mg/kg for food of plant origin, and 0.2 mg/kg for alcoholic beverages (except spirits), alcohol-free beverages, and herbal and fruit tea [49]. Italy regulation establishing the maximum allowable levels of total THC in hemp seeds is 2 mg/kg, hemp seed oil is 5 mg/kg, hemp seed flour is 2 mg/kg, and hemp-based supplements is 2 mg/kg [68]. Moreover, the state of Colorado permits up to 10 mg of THC per serving in edible products, reflecting a relatively permissive stance. Conversely, Oregon adopts a more conservative limit of 5 mg per serving. Similarly, Canada allows a maximum of 10 mg of THC per serving [61]. In Jamaica, THC content in food products ranges widely from 0.01 to 100 mg per 100 g [70].

Furthermore, a notable regulatory gap persists regarding the permissible concentrations of CBD, despite the rapid expansion of the global market for CBD-infused edibles. This segment alone was valued at approximately 2 billion dollars in 2022 and is projected to experience substantial growth, with estimates suggesting it will reach 6 billion dollars by the end of 2025 [71]. It is also worth noting that this regulatory gap persists despite public health advisories such as those issued by the FDA, which caution consumers, particularly pregnant women and children, against the use of CBD due to the lack of conclusive safety data and the potential for adverse effects [29,72].

In the United States, the legal framework surrounding *Cannabis* is particularly complex. Although numerous individual states have enacted legislation permitting the use of *Cannabis* for medicinal and, in some instances, recreational purposes, *Cannabis* remains classified as a Schedule I controlled substance under federal law. This inconsistency between state and federal regulations creates significant legal and regulatory inconsistencies [73]. Additionally, CBD has been officially classified as a medicinal product not only in the United States but also in several other jurisdictions, including Germany, Cyprus, and the United Kingdom. Consequently, CBD-containing products in these regions are subject to stringent regulatory requirements concerning safety, quality, and therapeutic efficacy, comparable to those governing pharmaceutical drugs [66]. However, a significant regulatory gap remains regarding whether hemp-derived oils, particularly those containing low levels of THC, fall under the same classification as medicinal products. In the Netherlands, for instance, a maximum threshold of 0.05% THC is tolerated in CBD products, despite the formal illegality of any detectable THC under Dutch narcotics legislation. This pragmatic stance acknowledges that even industrial hemp varieties inherently produce trace amounts of THC, making its complete exclusion from naturally derived CBD extracts technically challenging and often unfeasible [66].

Lastly, a significant regulatory consideration within the European Union pertains to the classification of *Cannabis*-derived food products under the Novel Food Regulation. Products such as hemp seeds, hemp seed oil, hemp flour, and defatted hemp seeds are not considered novel foods, as they have a well-documented history of consumption within the EU. Conversely, food products containing *Cannabis sativa* L. extracts, particularly those enriched with cannabidiol CBD, are classified as novel foods due to the absence of established consumption history in the region [60]. According to the EU Novel Food Catalogue, extracts with CBD concentrations exceeding those naturally present in the plant are deemed novel and, therefore, require comprehensive safety assessments prior to market authorization. However, regulatory ambiguity remains regarding whether hemp extracts that have not been enriched with isolated or synthetic CBD are subject to the same classification [66].

### 3.1. Hemp Oils

Currently, the market is flooded with numerous producers and sellers of hemp oils, and their numbers continue to grow rapidly. Despite the widespread availability of these products, significant uncertainties persist regarding their legal status, quality, and safety. Consequently, CBD remains under extensive examination for national health authorities, agricultural regulators, the World Health Organization (WHO), and the FDA. A key point of contention lies in determining the appropriate classification of CBD, whether it should be regarded as a food supplement, an investigational medicinal product, or even a controlled narcotic substance [66].

This regulatory ambiguity is further compounded by the variability introduced through diverse *Cannabis* cultivation practises, which can lead to significantly different hemp-derived extracts. Within the European Union, only hemp cultivars listed in the EU Common Catalogue of Agricultural Plant Species are legally permitted for cultivation, and these must not exceed a THC content of 0.2% *w/w* [28,50]. By contrast, other countries adopt more lenient standards. For instance, Canada permits up to 0.3% THC, while Switzerland allows up to 1% THC in hemp [66]. Globally, more than 700 documented *Cannabis* cultivars have been identified to date, primarily originating from *Cannabis Sativa*, *Cannabis Indica*, or hybrid strains. These cultivars display diverse morphotypic characteristics, which result in substantial variations in their phytochemical composition and, consequently, in the potency and therapeutic profile of the extracts derived from them [74]. Additionally, factors such as harvesting time, light exposure, photoperiod cycles, and soil pH have been shown to influence the phytochemical profile of the plant, particularly its phytocannabinoid content [22,75]. Furthermore, the specific plant parts used for extraction of the derived oil (flowering tops, leaves, stalks, or roots) greatly influences the final chemical composition mixture. Notably, the flowering tops of *Cannabis Sativa* typically contain the highest Δ^9^-THC concentrations, ranging from 10% to 12%, while the leaves contain approximately 1% to 2%, stalks 0.1% to 0.3%, and roots less than 0.03% [76,77].

In light of the above, hemp-derived oils are commonly classified into three main categories based on their cannabinoid composition. The first category, full-spectrum hemp oil, contains the complete array of naturally occurring cannabinoids including legally permissible amounts of Δ^9^-THC, as well as terpenes, flavonoids, and other phytonutrients. The second category, broad spectrum hemp oil, retains many of these cannabinoids and terpenes but specifically excludes Δ^9^-THC, making it more suitable for individuals concerned about psychoactive effects. The third category, CBD isolate, consists solely of purified CBD in the final product [78,79].

Nevertheless, a better and more clear to consumers classification approach may be beneficial. Specifically, evaluating hemp oil products according to their intended application, THC concentration, and the specific plant parts used during extraction could offer greater precision. Supporting this perspective, a recent study proposed an alternative classification system based on THC content, introducing two threshold models. Model A identifies hemp oil samples with THC concentrations exceeding 0.2% *w/w*, while Model B applies a slightly stricter criterion, flagging those surpassing 0.3% *w/w* [80]. Furthermore, depending on the intended use and the specific plant parts employed during the extraction process, hemp oils may be further classified into distinct types, such as hashish oil, essential oils, CBD oil, hemp vape oil, among others.

### 3.2. Hashish Oil

One of the most prevalent oils derived from the *Cannabis* plant is *Cannabis* oil, also referred to as hashish oil or hash oil. This highly potent THC oil is usually extracted from the flowers or buds of the *Cannabis* plant, which are rich in phytocannabinoids, as well as terpenoids and flavonoids. It can also be extracted from *Cannabis* resin, which is collected from the trichomes of the leaves [77]. It is also worth noting that fresh, undried plant material is less suitable for hash oil production, as much THC and CBD remains in their carboxylic acid forms THCA and cannabidiolic acid (CBDA), which may not be highly soluble in some organic solvents [81].

Notably, the THC content of hash oil can vary significantly, owing both to the diverse *Cannabis* varieties used and to the varying extraction methods employed by manufacturers. In addition, the form and concentration of THC in the final extract are contingent upon the extraction process employed, as well as the prevailing temperature and humidity conditions. Consequently, the extract may appear as a liquid, a clear yellowish-brown solid, a sticky semi-solid substance (commonly referred to as “wax”), or a brittle honeycombed solid (known as “honeycomb wax”) [82], with THC content as high as 90% [83].

The most common way to produce hash oil is through solvent extraction using chloroform, dichloromethane, petroleum ether, acetone, benzene, butane, methanol, ethanol, or isopropanol as solvent [84,85]. In particular, the extraction process involves placing *Cannabis* or *Cannabis* resin in a suitable vessel and adding an organic solvent at room temperature with continuous stirring. The extraction can be performed either passively or under reflux conditions. Once the extraction is complete, the mixture is filtered to separate the extracted material. Fresh batches of *Cannabis* material can then be introduced into the same vessel, utilizing the previously used solvent for subsequent extractions. This process can be repeated as necessary, allowing multiple batches to be processed using a single batch of solvent. Finally, the solvent is evaporated to achieve the desired consistency of the *Cannabis* oil, which has a strong herbal odor [77,86,87]. Other extraction techniques may involve maceration or infusion of different parts of the plant [28,88]. Nowadays, hash oil is frequently utilized for recreational purposes, medicinal purposes, for smoking or vaporizing, or incorporated into ready-to-consume products [77].

### 3.3. Hemp Seed Oil

Hemp seed oil is derived directly from the seeds of the *Cannabis* plant and contains only trace amounts of THC, but is rich in CBD, terpenoids, omega-6 and omega-3 essential fatty acids, and substantial amounts of γ-linolenic acid [87,89]. More precisely, linoleic and α- and γ-linolenic acid account for about 80% of its composition, along with polyphenols, γ-tocopherol, and vitamin E [90]. In addition, hemp seeds contain various oils (35.5%), proteins (24.8%), carbohydrates (27.6%), 5.4% digestible fibers, 22.2% non-digestible fibers, 6.5% moisture, and 5.6% ash [87]. All of these natural components may be present in hemp seed oil and contribute to its aroma and positive effects.

The process of obtaining hemp seed oil involves several essential steps to ensure the product’s quality and purity. Initially, mature hemp plants are harvested, and the seeds undergo meticulous cleaning and drying procedures to significantly reduce moisture content. This moisture reduction is critical to prevent mold growth and spoilage during subsequent storage and processing stages. Following these preparatory steps, two primary methodologies can be employed for oil extraction: cold pressing and solvent extraction [77]. In the cold pressing method, the cleaned and dried hemp seeds are mechanically processed to extract the oil. Typically, the seeds are first crushed or ground into a paste, and hydraulic presses are employed to exert pressure and extract the oil. This technique avoids any heat production, thereby preserving the nutritional properties and characteristic flavor of the oil [91].

Solvent extraction methods, such as Soxhlet extraction, ultrasound-assisted extraction, and microwave-assisted extraction, utilize solvents like hexane, methanol, or ethanol to dissolve oil from crushed hemp seeds [28,92,93]. After extraction, the solvent is filtered and evaporated, leaving behind pure hemp seed oil. However, this method may result in some loss of flavor and nutritional compounds due to the heat applied during solvent removal [91,94].

Alternatively, one of the most prominent extraction processes for hemp seed oil is supercritical CO_2_ extraction. This procedure involves cleaning and grinding the hemp seeds, setting up the extraction system with CO_2_, and maintaining a constant solvent flow rate. The method is highly effective due to its ability to preserve thermosensitive compounds, such as tocopherols and essential fatty acids, which might degrade at the higher temperatures used in other extraction procedures. Additionally, this process yields oil with higher concentrations of tocopherols compared to cold pressing and Soxhlet extraction, thereby enhancing the oil’s nutritional value and shelf life [95]. Another important advantage of the supercritical CO_2_ extraction technique lies in its classification as a “green” extraction method. Unlike conventional solvent-based techniques, supercritical CO_2_ leaves no toxic solvent residues in the final product, as the CO_2_ reverts to a gaseous state upon depressurization and dissipates entirely. This results in an exceptionally pure extract, free from chemical contaminants [95,96]. The concluding stage involves packaging the hemp seed oil in appropriate containers, often opting for dark-colored bottles to safeguard it from light exposure, as it may lead to the degradation of the oil’s quality over time [77].

Hemp seed oil is widely utilized today across numerous industries due to its rich nutritional profile and beneficial properties. It serves as a cooking oil and dietary supplement, enhancing diets with essential fatty acids, vitamins, and minerals [97,98,99]. In the cosmetics and skincare sector, it is valued for its moisturizing, anti-aging, and hair-strengthening properties, found in products like lotions, shampoos, and conditioners [100]. Additionally, it contributes to cardiovascular and immune system health in humans due to its omega-3 and omega-6 fatty acids, along with its anti-inflammatory, analgesic, anti-convulsant, and anti-psychotic properties [101,102,103].

### 3.4. Hemp Essential Oil

*Cannabis* essential oil, also known as hemp essential oil, is a volatile aromatic liquid extracted primarily from the flowers and upper leaves of the *Cannabis* plant. Unlike *Cannabis* oil, *Cannabis* essential oil contains only trace amounts of cannabinoids like THC and CBD. Instead, it is rich in terpenes, which are the compounds responsible for the plant’s distinctive aroma [77]. The essential oil can be obtained through hydrodistillation and steam distillation, both of which are traditional methods that utilize water or steam to extract the essential oil from plant materials. In hydrodistillation, the plant material is submerged in water and then heated to produce steam. As the water boils, the steam facilitates the release of essential oil from the plant cells. The mixture of steam and essential oil rises and passes through a cooling system where it condenses back into liquid form. A mixture of water and essential oil is then collected in a separating apparatus, where the essential oil is separated from the water due to differences in density. In steam distillation, steam is generated separately and then passed through the plant material. As the externally generated steam passes through the plant material, it disrupts the plant cells, releasing the essential oil. The steam, now carrying the essential oil, is then condensed back into liquid form through a cooling system. The resulting mixture of water and essential oil is collected and separated, similar to the process in hydrodistillation [104].

The chemical composition of hemp essential oil typically includes monoterpenes such as α-pinene, myrcene, and terpinolene, as well as sesquiterpenes like β-caryophyllene, humulene, and caryophyllene oxide. However, longer distillation times generally result in higher yields and more complex chemical profiles, including an increased concentration of cannabinoids and other valuable bioactive compounds [105]. Moreover, the concentration of those compounds can be significantly enhanced by pre-treatment procedures such as drying and microwave heating. For instance, exposing dry hemp inflorescences to microwave heating at 900 W for 1 min increases the abundance of bioactive compounds including CBD, (E)-caryophyllene, and caryophyllene oxide [104].

*Cannabis* essential oil is widely utilized in aromatherapy and alternative medicine. In aromatherapy, it is commonly used in diffusers to promote relaxation and alleviate stress, leveraging its soothing aromatic profile [106]. In addition, the distinctive aroma of *Cannabis* essential oil makes it a valuable component in natural perfume formulations and e-cigarette flavors, adding depth and character to various aromatic blends [107]. Furthermore, research has demonstrated that *Cannabis* essential oil can enhance sleep quality and is effectively employed in the alleviation of pain and stress [106,108,109,110].

### 3.5. CBD Oil

One of the most famous *Cannabis* products worldwide is CBD oil. This oil is a concentrated solvent extract from *Cannabis* flowers or leaves, which is then dissolved in an edible oil such as sunflower, hemp, or olive oil [66]. Consequently, it always contains at least a minimal amount of THC [111]. The solvents used in the extraction process can range from relatively harmless organic solvents like ethanol and isopropyl alcohol to more hazardous ones such as petroleum ether and naphtha, or even supercritical fluids like butane and CO_2_. During the extraction process, various plant components may be co-extracted with the desired cannabinoids from the herbal material. To remove these unwanted substances, a purification method known as “winterization” is commonly employed. This process entails freezing the extract at temperatures between –20 and –80 °C for 24 to 48 h. During this freezing period, substances with higher melting points, such as waxes, triglycerides, and chlorophyll, solidify and can be eliminated through filtration or centrifugation. This method considerably improves the flavor and appearance of the final product. However, the specific conditions and solvents used significantly impact the taste, color, and viscosity of the final product [66].

*Cannabis* oils exhibit varying concentrations of CBD, primarily influenced by the specific *Cannabis* variety utilized for extraction. Nevertheless, other variants, such as cannabigerol (CBG)-rich oil or THC-rich oil have also been observed, with additional types anticipated to emerge soon. Furthermore, the presence of terpenes in these oils is contingent upon the preparation method employed. Given their high volatility, terpenes may be significantly reduced when exposed to elevated temperatures during processes such as the drying of plant materials or the evaporation of solvents. However, it is feasible to capture the evaporated terpenes through condensation and reintroduce them into the final product [112,113]. Furthermore, additional ingredients may be incorporated to enhance characteristics such as color, viscosity, taste, or shelf life stability [66].

Users of CBD oil are drawn to its ability to deliver a concentrated dose of cannabinoids in an easily ingestible form, without the risk of intoxication associated with THC-rich products. This allows for the administration of significantly larger doses, enhancing its therapeutic potential [111]. Additionally, the dosage can be precisely managed by counting the number of drops consumed, providing a convenient and efficient method of administration. Furthermore, individuals who value the holistic approach of using herbal *Cannabis* often express concern of the distinctive odor produced by smoking or vaporizing it. In contrast, CBD oil is devoid of such odors, enabling discreet usage even in social settings such as workplaces or family gatherings [114].

Table 2 offers a concise yet comprehensive overview of the previously discussed categories of hemp-derived oil extracts, integrating additional information reported in the scientific literature. This includes their cannabinoid composition in CBD and THC and typical applications. Table 3 summarizes the principal bioactive compounds identified in hemp oils apart from cannabinoids, along with their associated biological effects. It is important to note that the concentration of these compounds can vary significantly depending on multiple factors, including the specific parts of the *Cannabis* plant used for extraction, the extraction technique employed, and the genetic strain or chemotype of the plant.

## 4. Medicinal *Cannabis*

In Europe, several *Cannabis*-based medicines have been authorized for clinical use as well, highlighting the increasing medical acceptance of cannabinoids. Sativex (Nabiximols), an oromucosal spray containing both THC and CBD, is approved for treating spasticity in multiple sclerosis. Similarly, synthetic THC formulations such as Marinol (Dronabinol) and Cesamet (Nabilone) are prescribed for cancer-related symptoms, AIDS-related anorexia, and nausea, while Bedrocan provides therapeutic *Cannabis* in the form of dried flower material [139]. Parallel developments have occurred in the United States, where Epidiolex, a purified CBD oral solution, received FDA approval in 2018 for the treatment of rare and severe childhood epilepsy syndromes such as Dravet and Lennox–Gastaut. These advancements reflect a growing body of pharmacological research on CBD that began in the 1970s and has accelerated in recent years due to discoveries related to the endocannabinoid system [140].

Moreover, an analysis of the CANNUSE database reveals that medicinal applications dominate recreational *Cannabis* use, accounting for 75.41% of all recorded cases, followed by psychoactive (8.35%) and nutrient (7.29%) purposes. Among the various plant parts utilized, leaves are the most frequently cited (50.51%), followed by seeds (15.38%) and inflorescences (11.35%). Leaf-based extracts have been traditionally used to address a range of health conditions, particularly digestive and nutritional disorders (17.66%), nervous system and mental health issues (16.24%), and pain and inflammatory conditions (12.21%). More specifically, the database includes 157 reports of *Cannabis* leaf use for gastrointestinal issues, 131 for neurological and psychological disorders, 108 for dermatological conditions, 105 for infections, and 101 for pain and inflammation [141].

### 4.1. The Endocannabinoid System

Numerous constituents derived from different parts of the *Cannabis* plant, such as terpenoids, flavonoids, stilbenes, alkaloids, and cannabinoids, have been scientifically validated for their diverse therapeutic properties [23,142,143]. In particular, many of these compounds have been demonstrated to interact with the human endocannabinoid system (ECS), a sophisticated cell-signaling network that includes two primary cannabinoid receptors, CB_1_ and CB_2_, which are part of the seven-transmembrane G-protein coupled receptor (GPCR) family [144]. These receptors play a crucial role in maintaining gastrointestinal balance, controlling food intake, managing visceral sensation, and regulating gastric secretion, as well as influencing pain perception, analgesia, stress response, emotional regulation, memory consolidation, anxiety relief, sleep regulation, and the overall maintenance of human homeostasis [27,145,146]. Alterations in the ECS have been demonstrated with various cancers but it remains unclear if these changes play a role in the malignant transformation of the cancer cells or are, rather, a consequence of the cancer being present [147].

CB_1_ receptors are located in various peripheral organs, including the heart, lungs, and prostate. Furthermore, they are present in the brain, particularly in regions such as the hippocampus, basal ganglia nuclei, cortex, and cerebellum. In these areas, they exhibit varying levels of expression, predominantly at neuron terminals, where they inhibit the release of neurotransmitters. Conversely, the CB_2_ receptor is abundantly expressed in several organs, including the lungs and testes, as well as in immune system organs and cells such as the spleen, thymus, tonsils, macrophages, and leukocytes [144,148].

Cannabinoids show substantial binding activity to CB receptors, functioning as either partial or complete agonists to endocannabinoid compounds. Remarkably, Δ^9^-THC functions as a partial agonist at both CB_1_ and CB_2_ receptors, while CBD demonstrates a low affinity for these receptors and acts as an inverse agonist at CB_2_ receptors [148,149]. In addition, it has been well established that CBD interacts with multiple molecular targets beyond the classical cannabinoid receptors. Notably, CBD acts as an antagonist of the GPR55 receptor, contributing to its anti-inflammatory and anticancer properties. It also functions as an agonist at the 5-HT1A serotonin receptor, thereby exerting analgesic and anxiolytic effects, partially through modulation of mu and sigma opioid receptors. Furthermore, CBD activates the TRPV-1 receptor, which plays a role in mediating its anti-inflammatory, pain-relieving, and sebum-regulating effects. Additionally, it enhances the activity of the adenosine A2A receptor by increasing extracellular adenosine levels, further amplifying its anti-inflammatory potential [150]. Other cannabinoids, such as cannabinol (CBN) and cannabigerol (CBG), exhibit different levels of affinity and activity to CB receptors, which contribute to the wide range of therapeutic effects of *Cannabis* [119]. Table 4 presents the binding activity for various major cannabinoids found in the *Cannabis* plant but also a comparison between CB1 and CB2 receptors.

### 4.2. Antioxidant and Anticancer Mechanisms

Upon activation, CB receptors initiate ion channels, the Mitogen-Activated Protein Kinase (MAPK) pathway and the adenylate cyclase (AC) pathway. The MAPK pathway, predominantly activated by CB_2_ receptors, plays a crucial role in regulating genes responsible for producing proteins involved in inflammation and apoptosis. Targeting this pathway has therapeutic potential in various cancer cell types, including breast, prostate, and liver cancers, thereby inhibiting tumor growth [155,156].

On the other hand, the AC pathway, activated by CB_1_ receptors, regulates the production of cyclic AMP (cAMP), a secondary messenger involved in many cellular processes, including cell proliferation and differentiation. In the context of cancer, modulating this pathway can impact tumor growth and progression [157]. The effects of cAMP in cell proliferation and survival are diverse and depend on cellular context. Phosphodiesterase (PDE) enzymes are responsible for converting cAMP to 5-AMP, thereby de-activating the downstream pathway [158]. It has been reported that increased cAMP levels caused by type IV PDE (PDE4) inhibitors affects HepG2 liver cancer cell cycle progression and survival [159]. Also, increasing cAMP levels has been shown to promote differentiation in various cancer cell types, including ovarian cancer cells, through the activation of PKA and downstream signaling pathways such as CREB [160].

Moreover, PDE4 inhibitor rolipram induces the expression of cell cycle inhibitors p21(Cip1) and p27(Kip1), resulting in growth inhibition, increased differentiation, and subsequent apoptosis of malignant cells [161]. On the other hand, cAMP can either promote apoptosis by activating the pro-apoptotic Bim protein or inhibit apoptosis via p-BAD and IAP-2 [162]. CB_1_ receptors can also modulate ion channels, specifically calcium and potassium channels. Calcium signaling is critical for various cellular functions, including cell cycle regulation, apoptosis, and metastasis. Potassium channels play a role in cell proliferation and apoptosis. By modulating these ion channels, cannabinoids can influence cancer cell survival and death [163].

Moreover, the composition of hemp oil and the relative concentrations of bioactive constituents plays a significant role in its chemopreventive and anticancer effects. Efficient decarboxylation of CBDA increases the concentration of CBD in hemp oil and leads to enhanced antioxidant activity and to significant anticancer effects in a variety of cell lines while being safe for normal fibroblasts [164]. Recent studies, elucidated the composition of extracts from hemp flowers and leaves as well as hemp oil by chemical profiling [165,166]. A total of 16 representative compounds, including CBD, cannabidivarin (CBDV), and CBN, were identified and their anticancer properties were characterized using metabolomics and proteomics; ultimately, hemp oil modified the expression of cyclin dependent kinases (CDKs), induced G1-phase arrest, and inhibited cell proliferation in colorectal cancer (CRC) cells [166].

#### 4.2.1. The Role of THC and CBD

As it was evidence throughout the literature, THC induces apoptosis in human leukemia cells via the CB_2_ receptor, with the inhibition of p38 MAPK being crucial for this apoptotic process [167]. Additionally, THC was shown to induce cytotoxicity in various leukemic cell lines through mechanisms independent of the CB_1_ and CB_2_ cannabinoid receptors. Notably, THC treatment leads to a significant decrease in the expression of phosphorylated ERK (pERK) protein, which is a key component of the MAPK pathway. Despite inducing apoptosis, THC does not significantly alter p53 protein levels [168]. P53 is responsible for inducing cell cycle arrest and apoptosis following DNA damage; in cancer, it is commonly mutated and inactive [169]. Therefore, agents that are able to induce apoptosis bypassing p53 activity are valuable in the fight against the disease.

Moreover, THC has been shown to significantly reduce cell viability and proliferation, while also inducing apoptosis in glioblastoma multiforme (GBM) cells under *in vitro* conditions [170]. *In vivo* studies using GBM xenograft models in both mice and rats demonstrated that THC treatment effectively suppresses tumor growth, providing promising preclinical evidence for its potential as an anti-glioma agent [171]. In addition, THC showed an immunomodulatory role by reducing cytokine production [172]. In a mouse model of CRC, THC inhibited the polarization of macrophages to the tumor promoting M2-like phenotype by targeting the SPP1/CD44 axis. Overall, THC inhibited proliferation, migration, and colony formation and promoted apoptosis in CRC cells [173].

**Figure 2:**
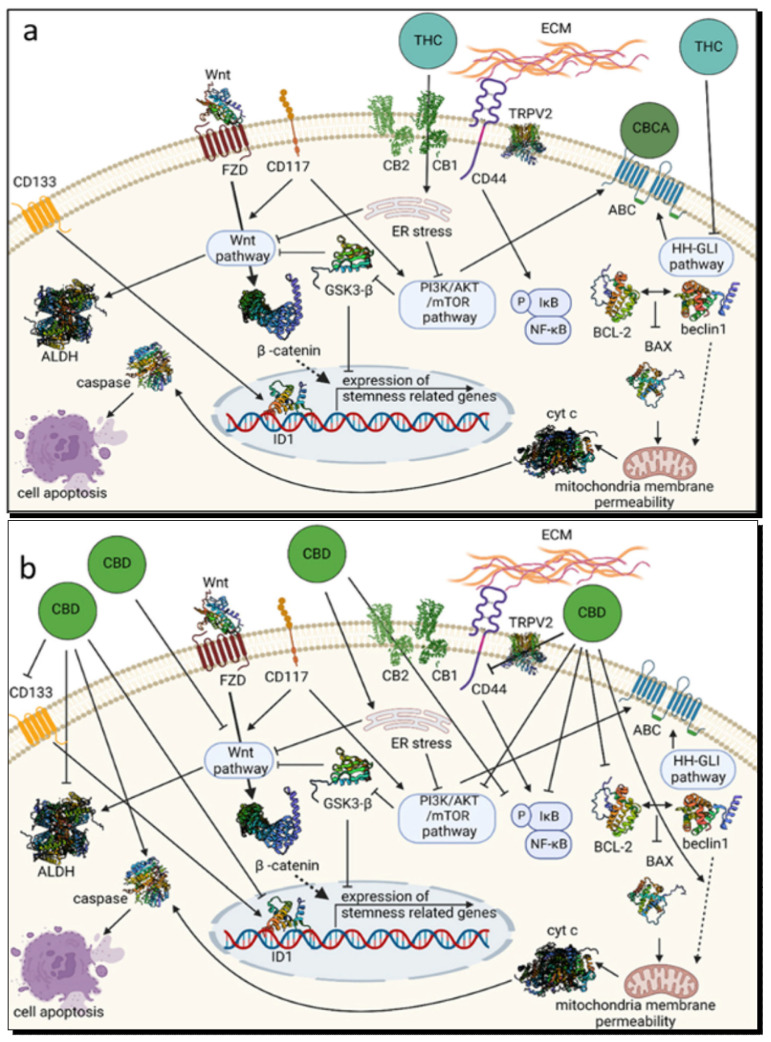
(**a**) A concise schematic of THC and CBCA anticancer mechanisms. ATP-binding cassette transporter; ALDH, aldehyde dehydrogenase; BCL-2, the activity of B-cell lymphoma-2; CB1, cannabinoid receptor type 1; CB2, cannabinoid receptor type 2; CBCA, cannabichromenic acid; CBD, cannabidiol; CD, clusters of differentiation; cyt c, cytochrome c; ECM, extracellular matrix; ER stress, endoplasmic reticulum stress; FZD, Wnt frizzled receptor; HH-GLI, Hedgehog-GLI; ID1, an inhibitor of DNA binding; TRPV2, transient receptor potential cation channel subfamily V member 2. (**b**) A concise schematic of CBD anticancer mechanism. CD133 (cluster of differentiation 133), ALDH (aldehyde dehydrogenase), FZD (frizzled receptor), CD117 (c-Kit), Wnt (Wingless-related integration site), CB1/CB2 (cannabinoid receptors 1 and 2), TRPV2 (transient receptor potential vanilloid 2), CD44 (cluster of differentiation 44), ABC (ATP-binding cassette), HH-GLI (Hedgehog–GLIoma) pathway, ER (endoplasmic reticulum) stress, PI3K (phosphoinositide 3-kinase), AKT, mTOR (mechanistic target of rapamycin) pathway, IκB (inhibitor of κB), NF-κB (nuclear factor κ-light-chain-enhancer of activated B-cells), BCL-2 (B-cell lymphoma 2), BAX (Bcl-2–associated X protein), ECM (extracellular matrix), GSK3-β (glycogen synthase kinase 3 β), ID1 (inhibitor of DNA binding 1) and cyt c (cytochrome c). Figure 2a,b, were taken with permission from Koltai et al. [144].

Figure 2a illustrates the multifaceted mechanisms through which THC exerts its effects on cancer cells, particularly by modulating pathways associated with cell survival, stemness, and apoptosis. Upon binding to CB1 and CB2 receptors, THC initiates intracellular signaling cascades that promote endoplasmic reticulum (ER) stress and influence key survival pathways, including the PI3K/AKT/mTOR and NF-κB pathways [170]. These interactions result in impaired cell proliferation and enhanced apoptotic responses. In addition, THC interferes with the Wnt/β-catenin pathway, notably through the inhibition of GSK3-β, which prevents the nuclear translocation of β-catenin and downregulates stemness-associated genes such as ID1. This disruption contributes to a reduction in the self-renewal capacity of cancer stem cells [144].

The pro-apoptotic effects of THC are further enhanced through increased mitochondrial membrane permeability, modulated by proteins such as Bax and Bcl-2, leading to the release of cytochrome c and activation of caspase-9 and effector caspases. THC also interacts with alternative receptor systems, such as CD44-TRPV2, which may modulate extracellular matrix dynamics and enhance cellular cytotoxicity. Moreover, the downregulation of stemness markers such as CD133, CD117, and ALDH further supports THC’s potential in targeting glioblastoma and other tumors with stem cell-like characteristics [144].

Likewise, CBD has been found to interact with cannabinoid receptors, as well as other receptors like TRPV1, (Peroxisome proliferator-activated receptor gamma) PPARγ, and serotonin 1A receptor (5-HT1A) to mediate its anticancer effects. In fact, CBD’s anticancer activity is both receptor-dependent and receptor-independent, involving mechanisms such as the induction of endoplasmic reticulum (ER) stress, autophagy, apoptosis, and the inhibition of key signaling pathways like PI3K/AKT/mTOR and MAPK/ERK [174]. CBD also binds to the often pro-tumorigenic G protein-coupled receptor 55 (GPR55), inhibiting the AKT pathway and leading to cell death [175]. In breast cancer, CBD reduces cell proliferation and induces apoptosis through the TRPV1 and PPAR-γ pathways, while also disrupting mitochondrial function, leading to cell death [174,176]. In lung cancer, CBD reduces cell viability and induces apoptosis through the cyclooxygenase-2 (COX-2) and PPAR pathways [177]. In gliomas, CBD induces apoptosis and autophagy by upregulating TRAIL/TRAIL-R2 signaling and affecting MAPK p38 and JNK pathways [178]. In leukemia and lymphoma, CBD mediates cell death through ROS production [174]. Additionally, in PC3 prostate cancer cells, CBD inhibits cell growth and induces apoptosis, partially mediated by CB2 receptors [179].

Moreover, a study has shown that CBD induces apoptosis and autophagy in head and neck cancer cells by activating DUSP1, which deactivates oncogenic MAPK signaling [180]. CBD significantly upregulated ataxia telangiectasia-mutated gene (ATM) and p53 protein levels in human gastric cancer cells and concurrently downregulated p21, leading to cell cycle arrest [181]. CBD has also demonstrated anti-angiogenic effects, inhibiting the formation of new blood vessels essential for tumor growth by downregulating molecules like MMP2, MMP9, ET-1, PDGF-AA, VEGF, and HIF-1α [174,182,183]. In lung cancer cell lines (A549, H538, and H460), CBD has been shown to reduce cellular invasiveness and promote the expression of ICAM-1 through the activation of the p42/44 MAPK pathway [184]. Similarly, in metastatic breast cancer cell lines (MDA-MB-231 and MDA-MB-436), CBD inhibited migration, invasiveness, and metastatic potential, an effect attributed to the downregulation of Id-1 protein expression via ERK modulation and reactive oxygen species (ROS) generation [185].

Beyond receptor modulation, CBD disrupts core molecular pathways essential for cancer cell survival and “stemness” such as Wnt/β-catenin, PI3K/AKT/mTOR, NF-κB, and Hedgehog. Specifically, Wnt/β-catenin promotes the expression of genes involved in uncontrolled proliferation and stem-like properties. By stabilizing the enzyme GSK-3β, CBD drives the degradation of β-catenin, thereby curbing the expression of pro-tumor genes such as ID1 [144,186]. However, this mechanism of CBD is yet to be shown in cancer cells.

The mechanism of CBD action is further presented in Figure 2b. CBD exerts its anticancer effects through a multi-targeted mechanism involving several cellular receptors and signaling pathways that regulate cell survival, stemness, and apoptosis [144,174]. Upon entering the tumor cell, CBD binds and activates PPARγ, a nuclear receptor, serving as a ligand. Once activated, PPARγ forms a heterodimer with RXR (Retinoid X Receptor), binds to PPREs (PPAR Response Elements) in DNA, and regulates transcription of target genes involved in cell cycle arrest, differentiation, and apoptosis. Activated PPARγ suppresses pro-survival genes like Bcl-2 and Bcl-xL and upregulates pro-apoptotic proteins such as Bax, Bak, and caspeases. This induces the mitochondrial apoptosis cascade. Reduced expression of these proteins disrupts mitochondrial integrity, making cells more sensitive to apoptotic stimuli. In addition, PPARγ activation indirectly inhibits PI3K/Akt, a major cell survival pathway. This increases sensitivity to apoptosis and inhibits cancer cell proliferation [187,188,189].

**Figure 3:**
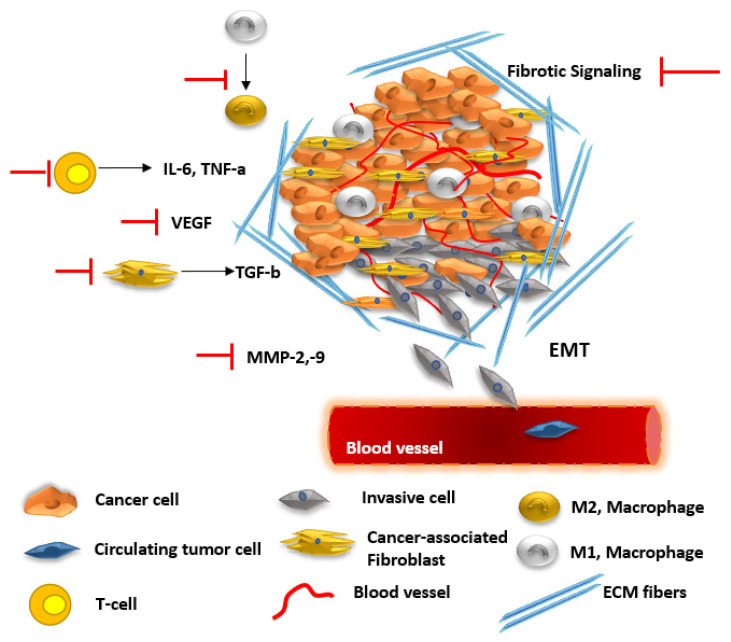
The effects of CBD and THC on the tumor microenvironment (TME). CBD suppresses pro-inflammatory cytokines (e.g., IL-6, TNF-α), helping reduce chronic inflammation associated with tumor progression. CBD also influences macrophage polarization by shifting towards the M1, antitumor phenotype (shifting M2→M1 phenotype). THC can also be immunosuppressive at high doses, reducing T cell activation and cytokine production. Both CBD and THC reduce angiogenesis by downregulating VEGF and MMPs. CBD impairs EMT and downregulates MMP-2/9, limiting cancer cell migration and invasion. Limited research suggests that cannabinoids may reduce fibrotic signaling and modulate stromal support for tumors, potentially altering tumor stiffness and invasiveness. Cancer-Associated Fibroblasts, CAFs; Cannabidiol, CBD; interleukin-6, IL-6; epithelial–mesenchymal transition, EMT; matrix metalloproteinases, MMPs; tumor necrosis factor alpha, TNF-α; Δ^9^-Tetrahydrocannabinol, THC; vascular endothelial growth factor, VEGF [190,191,192].

The anti-inflammatory properties of CBD also play a crucial role in modulating the tumor microenvironment (TME). The TME consists of different types of cells, including cells of the immune system and cancer-associated fibroblasts (CAFs), and other structural components, such as collagen and hyaluronan that create the ECM. The interactions between the TME and the tumor, as well as the presence of different cell types and structures, affect cancer cell growth, invasiveness, and response to treatment [190,191,192]. Modulation of the TME may increase drug influx to the tumor site by changing its desmoplasmic nature and increase immune cell infiltration [193]. Media containing natural compounds has been shown to restrict TME-regulated cell migration; treatment of breast cancer cells with CBD was shown to affect growth factor secretion to prevent lung fibroblasts from switching to the CAF-like phenotype [194,195]. A recent review highlighted the ability of CBD and other cannabinoids to modulate the tumor ECM [196]. TGF-beta plays a crucial role in TME remodeling and epithelial to mesenchymal transition (EMT) [197]. THC and CBD blocked the TGF-beta-mediated interaction between CAFs and cancer cells and inhibited the morphological changes associated with EMT [198]. Additionally, chronic inflammation is known to support tumor growth and progression, and by reducing inflammation, CBD can potentially decrease the supportive environment that tumors require for growth and metastasis [144]. Figure 3 further illustrates the complexity of the TME and highlights the inhibitory effects exerted by CBD and THC.

#### 4.2.2. Synergetic Effects

Current scientific research is also exploring the potential therapeutic and antioxidant properties of additional cannabinoids, including THCA, CBDA, CBG, cannabichromenic acid (CBCA), CBC, and THCV [27,154,199,200]. As an example, CBCA can influence apoptosis through distinct pathways, including the modulation of ABC transporters and inhibition of the HH-GLI pathway, converging on effectors like Beclin-1 and BAX [144]. Interestingly, THC and CBD demonstrated a synergistic effect in suppressing cell proliferation in GBM cell lines [201]. Furthermore, recent studies have revealed that CBG also diminishes GBM cell viability and invasiveness *in vitro* [202]. In addition, CBN was shown to act through ERK1/2 signaling, resulting in cell cycle arrest, suggesting a potential mechanism by which ECS modulation could suppress tumor progression in prostate cancer [203]. CBC has demonstrated significant anticancer activity against both breast and prostate cancer cell lines [204]. Moreover, it has been shown to act synergistically with THC, enhancing the inhibitory effects on bladder cancer cells [205].

Moreover, the synergetic effects of cannabinoids have shown potential in targeting cancer stem cells (CSCs), a subpopulation of tumor cells associated with tumor initiation, progression, recurrence, and resistance to therapy. CSCs are characterized by their high clonogenicity, tumorigenic potential, and capacity for self-renewal, making them a critical focus in the development of more effective cancer treatments [206]. This is particularly important as CSCs are often refractory to conventional therapies and are widely implicated in cancer relapse and metastatic spread. Recent reviews highlight the mechanism of action of CBD against cancer stem cells [171,207]. Notably, CBD downregulates the Wnt/β-catenin pathway, which is closely associated with drug resistance in CSCs, including those found in ovarian cancer (OCSCs) [144]. CBD has also been shown to enhance the activity of TRPV2, a protein involved in sensitizing cells to therapeutic agents [208]. CBD also plays a role in regulating Id1, a transcriptional regulator often abnormally expressed in CSCs. By modulating p38 MAPK signaling, CBD can indirectly influence other pro-tumorigenic pathways, such as STAT3 and TGF-β, contributing to a broader anticancer effect [209,210].

A recent study evaluated the synergistic effects of a 100:1 CBD:THC combination in various mouse models. The treatment alleviated light aversion in both male and female CD1 mice and reduced calcitonin gene-related peptide (CGRP) and SNP-induced squinting in males. In both FHM1 mutant and wild-type mice, while the treatment did not alter CSD characteristics, it reduced CSD-induced grimace responses, indicative of head pain relief. Importantly, no cognitive, emotional, or motor side effects were observed. The findings suggested that a CBD:THC combination may offer therapeutic benefits for certain migraine-associated symptoms without adverse effects [211]. In another study, evidence was presented for a potential synergistic interaction among THC, CBD, and CBDV on neuronal activity. When applied as a mixture, these cannabinoids produced a distinct modulation of hippocampal neuron firing compared to the effects observed when each compound was administered individually at the same concentrations. Notably, CBDV appeared to significantly counteract the combined effect of THC and CBD, highlighting the complex interplay among cannabinoids and their non-linear influence on neuronal network behavior [212]. Also, a formulation combining THC and CBD with temozolomide (TMZ) was investigated in preclinical models and subsequently assessed in a randomized, placebo-controlled phase II clinical trial in patients with GBM. The trial reported that the cohort receiving the THC:CBD and TMZ combination exhibited an increased one-year survival rate compared to the control group treated with TMZ monotherapy [170].

Moreover, it has been shown that THCA directly inhibits COX-1 and COX-2 enzymes, offering anti-inflammatory effects. Also, THCV acts as a CB1 and CB2 receptor antagonist at low doses but functions as a CB2 agonist at higher doses, potentially reducing inflammation and hyperalgesia. CBDA, the acidic form of CBD, inhibits COX enzymes, acts as a TRPV and TRPA agonist, blocks anandamide reuptake, and antagonizes 5-HT receptors [213]. Additionally, CBG interacts with both CB1 and CB2 receptors, functions as a potent α2-adrenoceptor agonist, antagonizes serotonin receptors, inhibits GABA reuptake, and modulates TRP channels, COX enzymes and lipoxygenase, indicating multifaceted therapeutic potential. Its precursor, cannabigerolic acid (CBGA), also inhibits COX-1 and COX-2 [214].

In another study, the co-treatment of etoposide with CBD was shown to significantly suppress autophagic flux and activate endoplasmic reticulum-associated degradation (ERAD) and unfolded protein response (UPR) signaling in LNCaP prostate cancer cells. The addition of CBD markedly enhanced etoposide-mediated inhibition of androgen receptor signaling, VEGF-A, the proto-oncogene c-Myc, and epithelial to mesenchymal transition. This combination therapy also induced apoptosis, as indicated by the activation of caspase-3 and cleavage of PARP-1. Moreover, co-administration substantially reduced tumorigenic features, including proliferative capacity, colony formation, cell migration, and 3D spheroid development, while promoting cellular senescence [215].

Cannabinoids may exert enhanced therapeutic effects through synergistic interactions not only with one another but also with a range of other bioactive compounds naturally present in the plant [27]. As an a example, stilbenes such as canniprene and cannastilbenes (I, IIa, and IIb), which are found in lower quantities compared to other phytochemicals in *Cannabis*, are believed to contribute meaningfully to the plant’s antioxidant defense system and may work synergistically with cannabinoids to enhance therapeutic outcomes [216].

Terpenes, such as β-caryophyllene, humulene, terpinolene, nerolidol, caryophyllene oxide, and borneol, are present in hemp oil products, providing their distinctive aroma and flavor [106]. In addition, β-caryophyllene is believed to interact with the endocannabinoid system through cannabinoid receptor CB_2_, exhibiting anti-inflammatory, analgesic, anxiolytic, and neuroprotective effects. However, unlike cannabinoids, most of the terpenes do not directly bind to CB_1_ and CB_2_ receptors. Instead, they modulate the effects of cannabinoids by influencing their receptor binding, a phenomenon known as the “entourage effect” [217]. Moreover, terpenes interact with various neurotransmitter receptors, enhancing the therapeutic effects of cannabinoids and contributing to the overall efficacy of *Cannabis* and hemp products. They engage with multiple biological targets, including cell membranes, enzymes, neurotransmitter receptors, muscle and neural ion channels, G protein-coupled receptors, and second messenger systems. Furthermore, they have been found to improve the body’s absorption of cannabinoids, potentially enhancing the analgesic properties of *Cannabis* [106,199].

Moreover, limonene has been shown to inhibit the production of NO, gamma-interferon, IL-4, and other pro-inflammatory cytokines, as well as lipopolysaccharide (LPS)-induced NO and PGE2. Additionally, when applied topically, limonene activates the TRPA1 channel, which may contribute to its anti-inflammatory action. Myrcene acts as an α2-adrenoreceptor agonist and modulates PGE2 levels to reduce inflammation. It also diminishes IL-1β-induced NO production and exhibits anti-catabolic effects that may slow the progression of osteoarthritis. Furthermore, myrcene possesses analgesic properties by interacting with opioid receptors in a manner reversible by naloxone [213]. α-Pinene exerts anti-inflammatory effects by inhibiting the production of PGE1, IL-1β, and IL-6 and reducing TNF-α expression in macrophages. Linalool, another prominent terpene, acts as an agonist of the adenosine A2A receptor and is believed to inhibit glutamate and substance P release at higher doses. It may also antagonize NK-1 receptors, contributing to its local anesthetic and anti-inflammatory effects [214]. Collectively, these terpenes enhance the therapeutic profile of hemp-derived products and support their potential use in managing inflammation and pain.

Flavonoids and alkaloids are also significant constituents of *Cannabis* with notable therapeutic potential. Flavonoids, such as apigenin, luteolin, quercetin, kaempferol, β-sitosterol, vitexin, isovitexin, and orientin, serve as pigments, provide UV protection, and act as signaling molecules [22]. Additionally, they have demonstrated antioxidant, anti-inflammatory, and neuroprotective activities [218,219]. Some flavonoids, such as vitexin and isovitexin, can bind to CB_1_ and CB_2_ receptors, contributing to their anti-inflammatory and analgesic effects [142]. Cannflavin A and B, which are unique to *Cannabis* sativa, are known inhibitors of prostaglandin E2 production by human rheumatoid synovial cells in culture [219]. Kaempferol acts as an anti-inflammatory agent for treating acute and chronic inflammation-induced diseases and has beneficial effects against cancer, obesity, diabetes, and vascular endothelial inflammation, as well as liver injury [2,17,220]. Quercetin, a polyphenolic flavonoid, demonstrates anticancer and anti-viral properties and is active against various microorganisms. It is also effective in treating allergic, metabolic, and inflammatory disorders [220,221,222]. Furthermore, alkaloids, including anabasine, scopolamine, and atropine, though present in trace amounts, add to the complex chemistry of *Cannabis* and have been recognized for their antibacterial and other pharmacological properties [223].

## 5. Hemp Oil Treatments

Despite promising advancements in cannabinoid research, there is a notable scarcity of studies specifically investigating the pharmaceutical potential of commercially available hemp oils for cancer treatment in humans and not animal models. A targeted search in the Scopus database spanning from 2000 to 2025 using the terms “hemp” AND “oil” initially retrieved 1883 publications. However, when refined to include “anticancer” and “antioxidant” as additional keywords, only four relevant studies emerged—published in 2021, 2023, and two in 2024. This limited number highlights a significant research gap and emphasizes the need for more focused investigations into the therapeutic efficacy and mechanistic pathways of hemp oils, particularly regarding their anticancer and antioxidant activities.

In a recent study conducted by Marciniuk J. et al. [224], 76 cold-pressed oil samples produced by nine Polish manufacturers were evaluated for their elemental composition, fatty acid profiles, antioxidant capacity, antimicrobial activity, and cytotoxicity. The findings revealed substantial variability in both composition and biological activity depending on the botanical origin and production methods of the oils. Among the samples analyzed, *Cannabis sativa* oil exhibited a notably high average total macronutrient content of 535.07 mg/kg, with individual values ranging from 268.83 to 938.55 mg/kg. It also contained the highest zinc concentration observed across all oils, reaching 8.79 mg/kg, and demonstrated manganese levels between 1.11 and 2.18 mg/kg in eight samples. The oil showed moderate to high antioxidant activity, attributed to its abundance of unsaturated fatty acids, polyphenols, and tocopherols. Its fatty acid composition, particularly rich in linoleic acid (omega-6) and α-linolenic acid (omega-3), supports known anti-inflammatory and cardioprotective effects. Furthermore, *Cannabis sativa* oil demonstrated antimicrobial efficacy against both Gram-positive and Gram-negative bacterial strains, as well as selected fungi. Importantly, cytotoxicity testing using the HFF-1 human fibroblast cell line confirmed that the oil was non-cytotoxic at tested concentrations, underscoring its potential safety for therapeutic or dietary use.

In colorectal cancer models, full-spectrum hemp oil extract inhibited the growth of tumor cells by arresting the cell cycle and altering metabolic pathways, both *in vitro* and *in vivo* [166]. Furthermore, long-term treatment with CBD isolate extract reduced the frequency of seizures and led to improvements in quality of life in children affected by refractory epilepsy [225,226]. In addition, *in vitro* assay demonstrated that full-spectrum hemp oil exerts pronounced antioxidant, antifungal, and anticancer effects over a concentration range of 15–300 µg/mL following a single 72 h exposure [227]. Lastly, a terpenocannabinoid-enriched hemp oil emulsion significantly accelerated wound healing and reduced skin inflammation without causing cutaneous toxicity [228].

Nevertheless, in a randomized controlled trial in maintenance hemodialysis patients, daily ingestion of 20 mL hemp seed oil failed to elicit significant reductions in the pro-inflammatory cytokines IL-6 or tumor TNF-α [229], indicating that its utility in managing systemic chronic inflammation may be limited to higher dosing, extended treatment duration, or co-administration with complementary anti-inflammatory agents. It is also worth mentioning that, in an earlier study by Chinello et al. [230], a case of cannabinoid poisoning in a child following a three-week period of hemp seed oil ingestion was reported. However, the worst adverse effects associated with CBD treatment are generally mild and well-tolerated, commonly including dizziness, dry mouth (xerostomia), gastrointestinal disturbances, and alterations in appetite [231]. Table 5 provides a concise overview of several clinical studies evaluating the therapeutic use of hemp oils so far.

## 6. Discussion and Future Directions

The available data so far concerning hemp oils present a mixed and contradictory picture. While several studies report promising anti-inflammatory, antioxidant, and anticancer properties associated with these products [166,227], others have raised concerns regarding their potential adverse effects. Notably, some reports have linked the use of certain hemp oils to unwanted outcomes such as muscle spasms, hallucinations [235], and mild cannabinoid poisoning [230].

Further clinical research is also needed to better understand CBD’s effects on hepatic enzymes, drug transporters, and its interactions with other medications, especially in cancer treatments [231]. A study investigating the potential synergy between CBD and other anticancer drugs, utilizing the Chou–Talalay method for combination index (CI) analysis, indicated that the co-administration of CBD with either cisplatin or paclitaxel exhibited antagonistic effects in both SK-OV-3 and OVCAR-3 ovarian cancer cell lines. However, mild synergism was observed in SK-OV-3 cells at high levels of growth inhibition [236]. Notably, priming SK-OV-3 cells with CBD or chemotherapeutic agents significantly enhanced drug sensitivity. These findings suggest that sequential, rather than simultaneous, administration of CBD with chemotherapy may enhance efficacy and potentially overcome drug resistance, though further *in vivo* studies are needed to confirm these results and clarify drug interactions

Although CBD, in its isolated form, or as part of pharmaceutical formulations such as Epidiolex, has been extensively studied and applied in various therapeutic settings, the current scientific evidence remains inadequate to verify comparable efficacy for commercially available hemp oils. It is also important to note that there is a significant lack of long-term, large-scale clinical studies evaluating the therapeutic use of hemp oils, leaving their chronic effects largely unknown.

Moreover, the growing public interest and widespread availability of hemp-derived oils raise important ethical concerns. In response, robust regulatory frameworks and standardized clinical guidelines should be established at the national level to safeguard consumer health and ensure product quality. Equally critical is the need to discourage individuals from self-administering these oils without appropriate medical oversight. Additionally, the use of CBD or THC-containing hemp products is strongly discouraged among vulnerable populations, particularly pregnant women and children [29]. Furthermore, greater awareness and regulatory clarity is needed regarding the appropriate classification of cannabis-derived oils, whether they should be treated as conventional food supplements or regulated as pharmaceutical products requiring medical prescription and supervision.

Although various analytical methodologies for cannabis testing have been developed and published, there is currently no universally accepted guideline yet. Consequently, cannabinoid content analyses may differ substantially across laboratories that use different methods of analysis, even when identical samples are tested, resulting in inconsistent data, potential consumer deception, and legal disputes [136]. In addition to this, a study conducted in the Netherlands demonstrated that 57% of tested cannabis oil samples contained THC concentrations exceeding 1%, with one sample reaching 57.5%. In contrast, other samples contained virtually no detectable cannabinoids, revealing significant discrepancies between labeled and actual content [237]. These inconsistencies were observed in both homemade and commercially available products.

Finally, consumers are strongly advised to avoid products such as hashish oil, which are typically rich in THC and associated with significant psychoactive effects and regulatory concerns. Instead, a preference should be given to CBD isolate oils. It is also important to note that scientific data regarding the composition, efficacy, and safety of other hemp-derived products, such as essential hemp oils and hemp vape oils, remain very limited. These formulations may involve distinct delivery methods and mechanisms of action, leading to different pharmacokinetic and pharmacodynamic behaviors compared to orally administered CBD oils. Consequently, further research is required to fully understand their therapeutic potential and associated risks.

## 7. Conclusions

Hemp oils contain a complex matrix of bioactive compounds including cannabinoids, terpenes, flavonoids, and fatty acids that act synergistically to exert antioxidant and anticancer effects. Mechanistic studies demonstrate their ability to reduce oxidative stress, induce apoptosis, inhibit angiogenesis, and target cancer stem-like cells. However, clinical translation is hindered by variability in composition, lack of standardized dosing, limited high-quality long-term trials, and ethical concerns surrounding unregulated use. Thus, while promising as adjunctive therapies, hemp oils require further clinical validation and regulatory oversight before integration into oncology practice.

## Figures and Tables

**Table 1:** The maximum allowable limits for THC in *Cannabis*-based edibles in general.

Country	Allowable Limit for THC	Reference
Germany	5 mg/kg	[47,62]
Canada	10 mg per package	[61]
Switzerland	20 mg/kg	[49]
United Kingdom	1 mg per product	[60,61]
Australia	2 mg per serving	[63]
Belgium	10 mg/kg	[64]
Cyprus	Zero tolerance	[65]
Netherlands	5 mg per product	[66]
United States	5–10 mg per serving	[58,67]
Italy	5 mg/kg	[68]
New Zealand	2 mg per serving	[69]
Jamaica	0.01–100 mg/100 g	[70]

**Table 2:** Information on the specific plant parts used for each product, reported THC and CBD levels, the extraction methods employed, and the corresponding shelf lives of each oil.

	Hemp Seed Oil	CBD Oil	Hashish Oil	Essential Oil	References
**Plant parts** **extracted**	Seeds (achene)	Aerial parts (flowers, leaves, sometimes stems)	Resinous parts of hemp (trichome-rich flowers/leaves), often processed into “hash” prior to oil extraction	Primarily flowers and leaves (terpene-rich plant material)	[77,98]
**THC levels** **CBD levels**	Negligible (<0.03%) * It can reach up to 20 *w/w*%	Up to legal limit (e.g., <0.3% *w/w*% in many jurisdictions) Usually 10–20 *w/w*% and sometimes even higher due to post-extraction enrichment	Variable; can be near legal limit (<0.3% for hemp-based) or higher if from non-hemp *Cannabis* ** 10–15 *w/w*%, but it can vary significantly	Trace quantities. Trace amounts (focus is on volatile terpenes rather than cannabinoids)	[59,89,98,115,116]
**Applications**	Edible food supplement and cosmetics	Medicinal uses and especially muscle relaxation, anti-inflammatory and antioxidant properties, cosmetics	Primarily for recreational use, some medicinal applications	Aromatherapy, perfumery, cosmetics	[23,28,56,89,117,118,119,120,121]
**Extraction** **approaches**	Cold-pressed or CO_2_ extraction, UAE, MAE and Soxhlet can also be used	CO_2_ extraction or SLE (organic solvent extraction utilizing petroleum ether, ethanol, methanol, acetone)	SLE (organic solvent extraction utilizing petroleum ether, ethanol, methanol, acetone)	Hydrodistillation and steam distillation	[91,93,94,104,122,123,124,125,126]
**Shelf life**	6–12 months	1–2 years, store in a cool place	1–2 years under airtight conditions	6–12 months	[127,128,129,130]

* Negligible THC content in hemp seed oil is typically due to contact with resinous plant material during harvesting/processing. ** In jurisdictions where non-hemp *Cannabis* is legal, “Hashish Oil” may contain significantly higher THC content and it can reach up to 90%.

**Table 3:** Major bioactive constituents in hemp oils other than cannabinoids and their typical concentrations and associated biological activities.

Category	Representative Compounds	Typical Content	Biological Activity	References
**Fatty acids**	Linoleic Acid (Omega-6)	50–60%	Skin barrier, anti-inflammatory	[87,89,97]
	α-Linolenic Acid (Omega-3)	15–25%	Cardiovascular, cognitive health	
	γ-Linolenic Acid (GLA)	~1–6%	Hormonal balance, eczema relief	
**Vitamins and antioxidants**	γ-Tocopherol (Vitamin E)	80–100 mg/100 g	Antioxidant, skin repair	[6,131]
	Phytosterols (β-sitosterol, campesterol)	~0.5–1%	Cholesterol-lowering, anti-inflammatory	
**Terpenes**	β-Caryophyllene, Myrcene, Limonene, Pinene, Linalool, Humulene	Trace < 0.5% (varies)	Entourage effect, anti-inflammatory, sedative, anxiolytic	[85,132,133]
**Aromatic compounds and additives**	Piperine, Curcumin, Cumarin, Coffee, other furans and pyrazines	Trace < 0.5% (added or infused)	Bioavailability enhancer, antioxidant, stimulant	[134,135]
	Stilbenes (e.g., canniprene, cannastilbenes)	Trace <0.5%	Antioxidant, anti-inflammatory, ECS synergy	
**Flavonoids and lignanamides**	Apigenin, Quercetin, Kaempferol, Cannflavins A/B *N*-caffeoyltyramine, grossamide, cannabisin B, cannabisin F	~0.01–3%	Anti-inflammatory, antioxidant, neuroprotective	[106,136,137]
**Carrier oil**	Sunflower Oil (*Helianthus Annuus*), Hemp Seed Oil (*Cannabis Sativa* L.), Olive Oil (*Olea Europaea*)	Usually is up to 90–99% of the oil product (9–9.9 mL of a 10 mL cbd oil bottle)	Skin hydration and barrier repair, contains antioxidant ingridients, improves cannabinoid solubility and stability, supports anti-inflammatory and cardiovascular health, enhances the synergistic effect when used with CBD	[47,66,87,138]

**Table 4:** Comparison of cannabinoid receptors (CB1 and CB2) and binding affinities of major cannabinoids [119,146,148,149,151,152,153,154].

CB1 Receptor	CB2 Receptor	Cannabinoid and Binding Activity to CB Receptors	
Primarily concentrated in the central nerve system and brain	Located primarily in immune cells and the peripheral nerve system	Δ^9^-THC	Partial agonist of CB1 and CB2 receptors
Responsible for psychoactive effects of cannabinoids	Not in charge of the psychoactive effects of cannabinoids	CBD	Low affinity for CB receptors and inverse agonist of CB2 receptor
Activation results in inhibition of neurotransmitter release	Activation results in inhibition of immune cell function and inflammatory response	CBN	Weak agonist for CB1 and higher affinity towards CB2 receptor
Plays a part in controlling hunger, mood, pain, and memory	Plays a part in controlling inflammation and immunological response	CBG	Low affinity for CB1 and CB2 receptors, but it affects the endocannabinoid system because of its ability to inhibit anandamide (AEA) uptake.
Associated with addiction and dependence on *Cannabis*	Has potential as a treatment for autoimmune disorders like multiple sclerosis	THCV	Partial agonist and in high doses, antagonist for CB1 and CB2 receptors
Highly expressed in regions including the cerebellum, basal ganglia, and hippocampus	Expressed at high levels in immune cells, such as B-cells and T cells	CBC	Low affinity for the cannabinoid receptors, but it affects the endocannabinoid system because of its ability to inhibit anandamide (AEA) uptake. It is also the most potent agonist of the transient receptor potential ankyrin subtype 1 protein (TRPA1)
Can be activated by endogenous cannabinoids, such as anandamide and 2-arachidonoylglycerol	Activated by endogenous cannabinoids, such as 2-arachidonoylglycerol, but less responsive to anandamide	Δ^8^-THC	Moderate partial agonistic effects on CB1 and CB2 receptors
Can be targeted by drugs that mimic or block cannabinoid activity	Can be targeted by drugs that modulate immune function and inflammation	CBDV	Very weak affinity for CB1 and CB2 receptors

**Table 5:** Summary of selected studies reporting benefits or drawbacks, dosage ranges, frequency of use, and type of hemp oil utilized in various clinical applications.

Benefit/Drawback	Dosage	Frequency	Type of Hemp Oil	Reference
Mild cannabinoid poisoning, including neurological symptoms (stupor, poor reactivity)	1 teaspoon (~5 mL)	Twice daily	CBD isolate	[230]
Reduce oxidative damage and cellular stress	10–40 mg	Daily	Full-spectrum	[164]
Reduce blood pressure and inflammation	600 mg (acute dose)	Single use or low daily dose	CBD isolate and full-spectrum	[232]
Anticancer effects via G1 cell cycle arrest and metabolic disruption in CRC	3–6 µg/mL (*in vitro*) 30 mg/kg (*in vivo*)	*In vitro*: 24–72 h *In vivo*: every 3 days	Full-spectrum	[166]
Antioxidant, antifungal, and anticancer activity	15–300 µg/mL (*in vitro*)	Single dose (72 h *in vitro*)	Full-spectrum	[227]
Anti-inflammatory effect in keratinocytes (reduce cytokines and improved skin barrier)	25–200 ng/mL (*in vitro*)	Single dose (24 h)	Hemp seed oil	[228]
Reduce inflammation, fibrosis, and necrosis and improve muscle function	10 mg/kg (*in vivo*) 18.75–300 µM (*in vitro*)	Daily for 14 days (*in vivo*)	Full-spectrum	[233]
Promising topical anti-inflammatory, analgesic, wound healing, and antioxidant effects	10–20% TC in hemp oil emulsifier	Daily for 14–28 days	Full-spectrum	[234]
No reduction in IL-6 or TNF-α in hemodialysis patients (does not decrease inflammation)	20 mL/day (3.68 g ALA)	Daily for 8 weeks	Hemp seed oil	[229]
Cause muscle spasms and hallucinations	Not specified	Single dose per day	Oil- extracts of *Cannabis* based on a 1:1 and 4:1 ratio of THC:CBD	[235]

## Data Availability

The data presented in this study are available in this article.

## Abbreviations

The following abbreviations are used in this manuscript:

ROS	Reactive Oxygen Species
RNS	Reactive Nitrogen Species
UV	Ultraviolet
NADPH	Nicotinamide Adenine Dinucleotide Phosphate (Reduced)
LOOH	Lipid Hydroperoxide
SOD	Superoxide Dismutase
CAT	Catalase
GPx	Glutathione Peroxidase
L-Arginine	L-Arginine
L-Citrulline	L-Citrulline
CBD	Cannabidiol
CBDA	Cannabidiolic Acid
CBDV	Cannabidivarin
Δ^9^-THC	Δ^9^-Tetrahydrocannabinol
THCA-A	Tetrahydrocannabinolic Acid A
THCA-B	Tetrahydrocannabinolic Acid B
CBN	Cannabinol
THCV	Tetrahydrocannabivarin
CBG	Cannabigerol
FDA	U.S. Food and Drug Administration
EFSA	European Food Safety Authority
BfR	Federal Institute for Risk Assessment
DNFI	Danish National Food Institute
WHO	World Health Organization
ARfD	Acute Reference Dose
bw	Body Weight
UB	Upper Bound
EU	European Union
SNP	Single Nucleotide Polymorphism
CYP	Cytochrome P450
8-OHdG	8-Hydroxy-2′-deoxyguanosine
BER	Base Excision Repair
NER	Nucleotide Excision Repair
OGG1	8-oxo-Guanine DNA Glycosylase
ASK1	Apoptosis Signal-Regulating Kinase 1
JNK	c-Jun N-terminal Kinase
p38	p38 Mitogen-Activated Protein Kinase
MAPK	Mitogen-Activated Protein Kinase
AC	Adenylate Cyclase
cAMP	Cyclic Adenosine Monophosphate
PDE	Phosphodiesterase
PDE4	Phosphodiesterase Type 4
PKA	Protein Kinase A
CREB	cAMP Response Element-Binding Protein
Bim	Bcl-2-interacting mediator of cell death
BAD	Bcl-2-associated Agonist of Cell Death
IAP-2	Inhibitor of Apoptosis Protein 2
p21(Cip1)	Cyclin-Dependent Kinase Inhibitor 1A
p27(Kip1)	Cyclin-Dependent Kinase Inhibitor 1B
ATP	Adenosine Triphosphate
5-AMP	5-Adenosine Monophosphate
TRPV1	Transient Receptor Potential Vanilloid 1
5-HT1A	5-Hydroxytryptamine Receptor 1A
GPR55	G-Protein Coupled Receptor 55
A2A	Adenosine A2A Receptor
CANNUSE	Cannabis Use Database
ECS	Endocannabinoid System
GPCR	G-Protein Coupled Receptor
CRC	Colorectal Cancer
CDK	Cyclin-Dependent Kinase
G1 Phase	Gap 1 Phase
Neutraceutical	Nutritional + Pharmaceutical
pERK	Phosphorylated Extracellular Signal-Regulated Kinase
p53	Tumor Protein p53
GBM	Glioblastoma Multiforme
SPP1	Secreted Phosphoprotein 1 (Osteopontin)
CD44	Cluster of Differentiation 44
ER	Endoplasmic Reticulum
PI3K	Phosphoinositide 3-Kinase
AKT	Protein Kinase B
mTOR	Mechanistic Target of Rapamycin
Wnt	Wingless/Integrated
Β-catenin	Beta-catenin
GSK3-Β	Glycogen Synthase Kinase 3 Beta
ID1	Inhibitor of DNA Binding 1
Bax	Bcl-2-associated X Protein
Bcl-2	B-cell Lymphoma 2
Caspase-9	Cysteine-aspartic Protease 9
CD44-TRPV2	CD44 and Transient Receptor Potential Vanilloid 2 Complex
CD133	Prominin-1
CD117	c-Kit
ALDH	Aldehyde Dehydrogenase
TRPV1	Transient Receptor Potential Vanilloid 1
PPARÎγ	Peroxisome Proliferator-Activated Receptor Gamma
5-HT1A	5-Hydroxytryptamine Receptor 1A
COX-2	Cyclooxygenase-2
TRAIL	TNF-related Apoptosis-Inducing Ligand
TRAIL-R2	TRAIL Receptor 2
DUSP1	Dual Specificity Phosphatase 1
ATM	Ataxia Telangiectasia Mutated
MMP2	Matrix Metalloproteinase 2
MMP9	Matrix Metalloproteinase 9
ET-1	Endothelin-1
PDGF-AA	Platelet-Derived Growth Factor AA
VEGF	Vascular Endothelial Growth Factor
HIF-1α	Hypoxia-Inducible Factor 1-alpha
ICAM-1	Intercellular Adhesion Molecule 1
Id-1	Inhibitor of DNA Binding 1
RXR	Retinoid X Receptor
PPREs	PPAR Response Elements
Bcl-xL	B-cell Lymphoma-extra Large
Bak	Bcl-2 homologous antagonist/killer
Caspases	Cysteine-aspartic Proteases
NF-κB	Nuclear Factor kappa-light-chain-enhancer of activated B-cells
Hedgehog	Hedgehog Signaling Pathway
A549	A549 Lung Carcinoma Cell Line
H538	H538 Lung Cancer Cell Line
H460	NCI-H460 Lung Cancer Cell Line
MDA-MB-231	MDA-MB-231 Breast Cancer Cell Line
MDA-MB-436	MDA-MB-436 Breast Cancer Cell Line
CD133	Cluster of Differentiation 133
FZD	Frizzled Receptor
ABC	ATP-Binding Cassette Transporters
IκB	Inhibitor of kappa B
TME	Tumor Microenvironment
CAFs	Cancer-Associated Fibroblasts
ECM	Extracellular Matrix
TGF-beta	Transforming Growth Factor Beta
EMT	Epithelial to Mesenchymal Transition
IL-6	Interleukin-6
TNF-α	Tumor Necrosis Factor Alpha
M1/M2	Macrophage Polarization States
THCA	Tetrahydrocannabinolic Acid
CBCA	Cannabichromenic Acid
CBC	Cannabichromene
CBDV	Cannabidivarin
CBGA	Cannabigerolic Acid
CGRP	Calcitonin Gene-Related Peptide
SNP	Sodium Nitroprusside
TMZ	Temozolomide
ERAD	Endoplasmic Reticulum-Associated Degradation
UPR	Unfolded Protein Response
PARP-1	Poly (ADP-ribose) Polymerase 1
Canniprene	Canniprene
Cannastilbenes	Cannastilbenes I, IIa, IIb
β-Caryophyllene	Beta-Caryophyllene
